# 

**Published:** 1861-09

**Authors:** 


### ORIGINAL COMMUNICATIONS.

PAGE.
ART. I.—Rhus Texicodendron—The Cause of Milk Sickness. By E. C. Landrum, M. D., Columbus, Miss. .... 1
ART. II.—Letter from Richmond. By Dr. J. G. Westmoreland. .... 5

### SELECTIONS.

Treatment of Delirium Tremens by Digitalis. By G. M. Jones, Esq., Dr. Williams, and Dr. Webster. .... 9
On the Theory of Simple Epilepsy. By Dr. Chas. Bland Radcliffe, Physician to the Westminster Hospital. .... 16
Chlorate of Potash in Phthisis—Notes of three cases treated with Chlorate of Potash, in the Buffals General Hospital. By C. C. F. Gay, M. D., Attendidg Physician, and Reported by J. A. Peters, Med. Student. .... 30
Report of Cases of Phthisis, Scrofula, and other diseases, treated by the Chlorate of Potash. By E. G. Fountain, A. M., M. D., of Davenport, Iowa. .... 32
Coma and Narcotism. .... 47
Croup—Quinine. .... 48
Wounds. .... 51
On the Resorption of Pleuritic Exudations. By Prof. Skoda. .... 56

### EDITORIAL AND MISCELLANEOUS.

Medical Department of our Army. .... 58

Caffeine in Opium—Coma. .... 61

Camphor—Poisoned by. .... 62

